# Diagnostic Classification Models for Ordinal Item Responses

**DOI:** 10.3389/fpsyg.2018.02512

**Published:** 2018-12-11

**Authors:** Ren Liu, Zhehan Jiang

**Affiliations:** ^1^Psychological Sciences, University of California, Merced, Merced, CA, United States; ^2^University Libraries, University of Alabama, Tuscaloosa, AL, United States

**Keywords:** diagnostic classification model, ordinal item responses, partial credit model, rating scales, Bayesian estimation, Markov Chain Monte Carlo (MCMC)

## Abstract

The purpose of this study is to develop and evaluate two diagnostic classification models (DCMs) for scoring ordinal item data. We first applied the proposed models to an operational dataset and compared their performance to an epitome of current polytomous DCMs in which the ordered data structure is ignored. Findings suggest that the much more parsimonious models that we proposed performed similarly to the current polytomous DCMs and offered useful item-level information in addition to option-level information. We then performed a small simulation study using the applied study condition and demonstrated that the proposed models can provide unbiased parameter estimates and correctly classify individuals. In practice, the proposed models can accommodate much smaller sample sizes than current polytomous DCMs and thus prove useful in many small-scale testing scenarios.

Grouping people into different categories are often of interest in educational and psychological tests. For example, the Five Factor Personality Inventory-Children (McGhee et al., 2007) aims to identify which personalities a child possesses. In another case of career assessment, the Strong Interest Inventory (Prince, 1998; Staggs, 2004; Blackwell and Case, 2008) aims to categorize individuals into occupational themes for identifying their career interest areas. From a psychometric standpoint, those tests share at least three commonalities. First, they are usually multidimensional tests, meaning that multiple latent traits are assessed. Second, the purpose of such tests is to label individuals through assigning them with one of the pre-defined categories. Third, they usually allow for ordinal item responses such as strongly disagree, disagree, agree and strongly agree. For scoring tests with such features, diagnostic classification models (DCMs) have provided an attractive framework in psychometrics because they are designed to classify individuals into pre-defined latent categories (Rupp and Templin, 2008; Rupp et al., 2010). However, most current DCMs for polytomous items consider item response categories as nominal without using the ordered category information (Templin et al., 2008; e.g., de la Torre, 2010; Ma and de la Torre, 2016). As a result, those models are often large and require a sample size hardly attainable for parameter estimation. The purpose of this study is to create smaller ordinal DCMs that are designed to score individuals on an ordinal scale. In this article, we first review current polytomous DCMs. Then, we explain the theoretical development of the proposed models. Next, we fit the proposed models to an operation dataset and compare their performance with a current polytomous DCM in which the ordered structure is ignored. Afterwards, we performed a small simulation study using the applied study condition to evaluate the parameter recovery of the proposed models. Finally, we discuss the application and advantages of the models and offer future research recommendations.

## Review of Current Polytomous DCMs

Existing literature has considered DCMs from either the perspective of Bayesian networks or confirmatory latent class models. In the Bayesian networks literature, the Dibello-Samejima modeling framework advanced by Almond et al. (2001, 2009, 2015), and Levy and Mislevy (2004) is an example of scoring polytomous item data. In this article, we consider DCMs as confirmatory latent class models with two outstanding features. First, the latent traits, commonly referred to as attributes, are defined *a priori*. The possible possession status of all latent traits forms latent classes, commonly referred to as attribute profiles. In this article, we use *k* = 1, …, *K* to index latent traits and **α**_*c*_ = {α_1_, …, α_*K*_} to index attribute profiles for latent class *c*. Second, the measurement relationship between items and attributes is defined *a priori*. This information is contained in an item-by-attribute incidence matrix, commonly referred to as the Q-matrix (Tatsuoka, 1983), where an entry *q*_*ik*_ = 1 when item *i* measures attribute *k*, and *q*_*ik*_ = 0 otherwise.

To our knowledge, eight DCMs have been developed to score polytomous item data. Each model is constructed through applying a polytomous extension method to a dichotomous DCM. We listed such information in Table 1. Most polytomous DCMs are developed based on the log-linear cognitive diagnosis model (LCDM; Henson et al., 2009) or its equivalent: the generalized deterministic input noisy “and” gate (GDINA; de la Torre, 2011) model. The NRDM, GDM, PC-DINA, and SG-DINA utilize the concept of the nominal response model (NRM; Bock, 1972) in item response theory where each response option in each item has its own intercept and slope; The P-LCDM, DINA-GD, and GPDM utilize the concept of the graded response model (GRM; Samejima, 1969) where the differences between cumulative probabilities of adjacent options are modeled; the RSDM utilize the concept of the rating scale model (RSM; Andrich, 1978) where items measuring the same set of attributes share response option parameters. To summarize, many current DCMs are built to accommodate nominal response data. For example, the NRDM defines the probability of individuals in latent class *c* selecting response option *m* on item *i*, such that

(1) $$P(X_{i}=m|\alpha_{c})=\frac{\exp[\lambda_{0,i,m}+\lambda_{i,m}^{T}h\left(\alpha_{c},q_{i}\right)]}{\sum_{m=0}^{M-1}\exp[\lambda_{0,i,m}+\lambda_{i,m}^{T}h\left(\alpha_{c},q_{i}\right)]},$$

where λ_0, *i, m*_ is the intercept parameter associated with option *m* on item *i*, and $\lambda_{i,m}^{T}h\left(\alpha_{c},q_{i}\right)$ index all the main effects and higher-order interaction effects of the *k* attributes associated with option *m* on item *i*, which can be expressed as $\sum_{k=1}^{k}\lambda_{1,i,k,m}\left(\alpha_{c,k}q_{i,k}\right)+\sum_{k=1}^{k-1}\sum_{k^{\prime }=k+1}^{K}\lambda_{2,i,k,k^{\prime },m}\left(\alpha_{c,k}\alpha_{c,k^{\prime }}q_{i,k}q_{i,k^{\prime }}\right)+.$ ···

**Table 1:** Previous DCMs for scoring polytomous item data.

**Model**	**Full Name**	**Dichotomous Core**	**Similar Extension Method in IRT Models**
NRDM	The nominal response diagnostic model (Templin et al., 2008)	LCDM	The nominal response model (NRM; Bock, 1972)
GDM	The general diagnostic model (von Davier, 2008)	LCDM	NRM
PC-DINA	The partial-credit deterministic input noisy “and” gate model (de la Torre, 2010)	DINA	NRM
P-LCDM	The polytomous log-linear cognitive diagnosis model (Hansen, 2013)	LCDM	The graded response model (GRM; Samejima, 1969)
PC-DINA	The sequential generalized DINA model (Ma and de la Torre, 2016)	LCDM	NRM
DINA-GD	The DINA model for graded data (Tu et al., 2017)	DINA	GRM
GPDM	The general polytomous diagnosis model (Chen and de la Torre, 2018)	LCDM	GRM
RSDM	The rating scale diagnostic model (Liu and Jiang, submitted)	LCDM	The rating scale model (RSM; Andrich, 1978)

Let us break down the summation symbol in Equation 1 for an instructional example. On item *i* with four response options (*M* = 4): 0,1,2, and 3, the probability of selecting response option 2 is expressed as

(2) $$P\left(X_{i}=2|\alpha_{c}\right)=\frac{\exp[\lambda_{0,i,2}+\lambda_{i,2}^{T}h\left(\alpha_{c},q_{i}\right)]}{\begin{array}{c} \exp[\lambda_{0,i,0}+\lambda_{i,0}^{T}h\left(\alpha_{c},q_{i}\right)]+\\ \exp\left[\lambda_{0,i,1}+\lambda_{i,1}^{T}h\left(\alpha_{c},q_{i}\right)\right]+\\ \exp\left[\lambda_{0,i,2}+\lambda_{i,2}^{T}h\left(\alpha_{c},q_{i}\right)\right]+\\ \exp[\lambda_{0,i,3}+\lambda_{i,3}^{T}h\left(\alpha_{c},q_{i}\right)] \end{array}}.$$

It should be clear in Equation 2 that each option in item *i* is associated with its own set of intercept, main effects and higher-order interaction parameters. As a result, the NRDM is able to accommodate polytomous response options that can be either ordered or not ordered.

## Model Development

To develop DCMs that utilize the ordered structure of response options in many polytomous items (e.g., 0 = never, 1 = seldom, 2 = sometimes, 3 = usually), we contemplated on how the parameters on the NRM can be constrained to create the Generalized Partial Credit Model (GPCM; Muraki, 1992) and Generalized Rating Scale Model (GRSM; Muraki, 1992) in item response theory. The probability of selecting option *m* on item *i* given a unidimensional latent trait θ for examinee *e* is defined as

(3) $$P(X_{i}=m|\theta_{e})=\frac{\exp\left(d_{im}\theta_{e}+b_{im}\right)}{\sum_{m=0}^{M-1}\exp\left(d_{im}\theta_{e}+b_{im}\right)},$$

for the NRM,

(4) $$P(X_{i}=m|\theta_{e})=\frac{\exp\sum_{m=0}^{m}\left[d_{i}\left(\theta_{e}+b_{im}\right)\right]}{\sum_{s}^{M-1}\exp\sum_{m=0}^{s}\left[d_{i}\left(\theta_{e}+b_{im}\right)\right]},$$

for the GPCM, and

(5) $$P(X_{i}=m|\theta_{e})=\frac{\exp\sum_{m=0}^{m}\left[d_{i}\left(\theta_{e}+b_{i}+t_{m}\right)\right]}{\sum_{s}^{M-1}\exp\sum_{m=0}^{s}\left[d_{i}\left(\theta_{e}+b_{i}+t_{m}\right)\right]},$$

for the GRSM. The *d*_*im*_ and *b*_*im*_ in Equation 3 are the slope parameter and intercept parameter for option *m* in item *i*, respectively. In Equation 4, the slope parameter *d*_*i*_ loses the subscript *m*; instead, summation symbols are used such that the *d*_*im*_ in Equation 3 is represented by *m*×*d*_*i*_ ∀ *m*>0 in Equation 4. To obtain Equation 5, an extra constraint is imposed on Equation 4 where the *b*_*im*_ is decomposed into a general item intercept for item *i*: *b*_*i*_ and a general response option intercept for option *m*: *t*_*m*_ that is applicable to all items.

Inspired by how the NRM can be constrained to arrive at the GPCM and GRSM, we propose two ordinal DCMs through applying constraints to the NRDM so that the proposed models are targeted for scoring ordered item data. We refer to these models as the Ordinal Response Diagnostic Model (ORDM) and the Modified Ordinal Response Diagnostic Model (MORDM). The ORDM is defined as

(6) $$P(X_{i}=m|\alpha_{c})=\frac{\exp\sum_{m=0}^{m}\left[\lambda_{0,i,m}+\lambda_{i}^{T}h\left(\alpha_{c},q_{i}\right)\right]}{\sum_{s}^{M-1}\exp\sum_{m=0}^{s}\left[\lambda_{0,i,m}+\lambda_{i}^{T}h\left(\alpha_{c},q_{i}\right)\right]},$$

where $\lambda_{i}^{T}h\left(\alpha_{c},q_{i}\right)\;=\;\sum_{k=1}^{K}\lambda_{1,i,k}(\alpha_{c,k}q_{i,k})\;+\;\sum_{k=1}^{K-1}\sum_{k^{\prime }=k+1}^{K}\lambda_{2,i,k,k^{\prime }}(\alpha_{c,k}\alpha_{c,k^{\prime }}q_{i,k}q_{i,k^{\prime }})+..\;..$ For identifiability purposes, we impose three sets of constraints on the ORDM. First, in order to fix the scale, we adopt Thissen (1991)'s approach and fix all parameters associated with the first response option to 0, such that

$$\sum_{m=0}^{0}\left(\lambda_{0,i,m}\right)=0\;\forall \;i,$$

$$\sum_{m=0}^{0}\left(\lambda_{1,i,k}\right)=0\;\forall \;i,\;k,$$

$$\sum_{m=0}^{0}\left(\lambda_{2,i,k,k^{\prime }}\right)=0\;\forall \;i,k,k^{\prime },$$

and for all higher-order interactions. Second, we constrain parameters associated with main effects and higher-order interactions to be < 0 so that the possession of more attributes increases the probability of selecting a higher response option:

$$\lambda_{1,i,k}>0\;\forall \;k,$$

$$\lambda_{2,i,k,k^{\prime }}>0\;\forall \;k,k^{\prime },$$

and for other higher-order interactions. Third, we constrain intercept parameters of a higher response option to be smaller than those of a lower response option so that the probability of selecting a higher response option is smaller for individuals without the measured attributes such that

$$\lambda_{0,i,m}\geq \lambda_{0,i,m+1}\forall \;i,m.$$

Comparing Equation 6 to Equation 1, the $\lambda_{i,m}^{T}$ in Equation 1 loses the subscript *m*. The **λ**_**i**_ parameters in Equation 6 are summated for their associated response options.

Let us break down the summation symbol in Equation 6 for an instructional example. On item *i* with four response options: 0,1,2, and 3, the probability of selecting response option 2 is expressed as

(7) $$P(X_{i}=2|\alpha_{c})=\frac{\exp\left[0+\left[\lambda_{0,i,1}+\lambda_{i}^{T}h\left(\alpha_{c},q_{i}\right)\right]+\left[\lambda_{0,i,2}+\lambda_{i}^{T}h\left(\alpha_{c},q_{i}\right)\right]\right]}{\begin{array}{l} \exp\left(0\right)+\exp\left[0+\left[\lambda_{0,i,1}+\lambda_{i}^{T}h\left(\alpha_{c},q_{i}\right)\right]\right]+\exp[0+\left[\lambda_{0,i,1}+\lambda_{i}^{T}h\left(\alpha_{c},q_{i}\right)\right]+\left[\lambda_{0,i,2}+\lambda_{i}^{T}h\left(\alpha_{c},q_{i}\right)\right]]+\\ \;\exp[0+\left[\lambda_{0,i,1}+\lambda_{i}^{T}h\left(\alpha_{c},q_{i}\right)\right]+\left[\lambda_{0,i,2}+\lambda_{i}^{T}h\left(\alpha_{c},q_{i}\right)\right]+\left[\lambda_{0,i,3}+\lambda_{i}^{T}h\left(\alpha_{c},q_{i}\right)\right]] \end{array}}$$

Equation 7 is similar to Equation 2 in two ways. First, both equations ask what the probability is that an individual in latent class *c* selecting option 2 as compared to the sum of probabilities of all response options that the individual could select. Second, the intercept parameter is freely estimated for each response option in each item (e.g., λ_0, *i*, 1_, λ_0, *i*, 2_, and λ_0, *i*, 3_). However, what is different is that the $\lambda_{i,2}^{T}h\left(\alpha_{c},q_{i}\right)$ in Equation 2 is replaced by $2\times \lambda_{i}^{T}h\left(\alpha_{c},q_{i}\right)$ in Equation 7. It should be clear now that the proposed ORDM can be expressed as a constrained version of the NRDM, analogous to how the GPCM can be formulated as a constrained version of the NRM.

The MORDM is defined the same as the ORDM in Equation 6, except that the λ_0, *i, m*_ is decomposed into general item parameters and shared response option parameters. Before deciding to share response option parameters across all items, we should remember that DCMs are multidimensional models while the NRM is a unidimensional model. Therefore, it would be unwarranted to assume that all items in a DCM can share the same set of response option parameters because those items may measure different traits. Instead, what we can do is to allow response option parameters to be shared within each dimension. As introduced above, DCMs are confirmatory latent class models, which means that the dimensions in DCMs can be represented through latent classes (i.e., attribute profiles). We express the relationship between items and attribute profiles in an item-by-attribute-profile incidence matrix called the W-matrix (Liu and Jiang, submitted), where an entry *w*_*iv*_ = 1 when item *i* measures attribute set *v*, and 0 otherwise. By definition, each column corresponds to a unique attribute profile; each row has only one entry of 1 and all others of 0. Utilizing the W-matrix, we are able to allow response option parameters to be shared within items that measure the same set of attributes. Subsequently, the λ_0, *i, m*_ in Equation 6 is decomposed into λ_0, *i*_ and $\sum_{v=1}^{V}\lambda_{0,m_{v}}w_{iv}$ in the MORDM, where the $\sum_{v=1}^{V}\lambda_{0,m_{v}}w_{iv}$ represents the response option parameters shared across items that measure attribute set *v*. Now, we can define the MORDM as

(8) $$P(X_{i}=m|\alpha_{c})=\frac{\exp\sum_{m=0}^{m}[\lambda_{0,i}+\sum_{v=1}^{V}\lambda_{0,m_{v}}w_{iv}+\lambda_{i}^{T}h\left(\alpha_{c},q_{i}\right)]}{\sum_{s}^{M-1}\exp\sum_{m=0}^{s}[\lambda_{0,i}+\sum_{v=1}^{V}\lambda_{0,m_{v}}w_{iv}+\lambda_{i}^{T}h\left(\alpha_{c},q_{i}\right)]}.$$

The constraints we impose on the MORDM is the same as those on the ORDM, except that the third constraint (i.e., for the intercept parameters) needs to be adapted to the MORDM. In the MORDM, we impose this constraint:

$$\sum_{v=1}^{V}\lambda_{0,m_{v}}w_{iv}\leq \sum_{v=1}^{V}\lambda_{0,m-1_{v}}w_{iv}\forall \;v,m.$$

to make sure that individuals without the measured attributes have a smaller probability of selecting a higher response option.

Let us continue the example of selecting response option 2 on an item with options 0,1,2, and 3. The MORDM in such case is expressed as

(9) $$\begin{array}{l} P\left(X_{i}=2|\alpha_{c}\right)=\frac{\exp\left[0+\left[\lambda_{0,i}+\sum_{v=1}^{V}\lambda_{0,m=1_{v}}w_{iv}+\lambda_{i}^{T}h\left(\alpha_{c},q_{i}\right)\right]+\left[\lambda_{0,i}+\sum_{v=1}^{V}\lambda_{0,m=2_{v}}w_{iv}+\lambda_{i}^{T}h\left(\alpha_{c},q_{i}\right)\;\right]\right]}{\exp\left(0\right)+\exp\left[0+\left[\lambda_{0,i}+\sum_{v=1}^{V}\lambda_{0,m=1_{v}}w_{iv}+\lambda_{i}^{T}h\left(\alpha_{c},q_{i}\right)\right]\right]+}\\ \;\exp\left[0+\left[\lambda_{0,i}+\sum_{v=1}^{V}\lambda_{0,m=1_{v}}w_{iv}+\lambda_{i}^{T}h\left(\alpha_{c},q_{i}\right)\right]+\left[\lambda_{0,i}+\sum_{v=1}^{V}\lambda_{0,m=2v}w_{iv}+\lambda_{i}^{T}h\left(\alpha_{c},q_{i}\right)\right]\right]+\\ \;\exp[0+\left[\lambda_{0,i}+\sum_{v=1}^{V}\lambda_{0,m=1_{v}}w_{iv}+\lambda_{i}^{T}h\left(\alpha_{c},q_{i}\right)\right]+\left[\lambda_{0,i}+\sum_{v=1}^{V}\lambda_{0,m=2_{v}}w_{iv}+\lambda_{i}^{T}h\left(\alpha_{c},q_{i}\right)\right]\\ \;+\left[\lambda_{0,i}+\sum_{v=1}^{V}\lambda_{0,m=3_{v}}w_{iv}+\lambda_{i}^{T}h\left(\alpha_{c},q_{i}\right)\right]] \end{array}$$

Equation 9 can be viewed as a constrained version of Equation 7 where the intercept parameters are decomposed. To summarize, one can constrain the main effect parameters of the NRDM to arrive at the ORDM, and further constrain the intercept parameters of the ORDM to arrive at the MORDM.

## Operational Study

The purpose of this operational study is to compare the performance of the ORDM and the MORDM with the NRDM through fitting these three models to an ordinal item response dataset. The motivating research question was: can the more parsimonious ORDM and/or the MORDM perform similarly to the NRDM? To answer this question, we looked into the following six types of outcomes: (1) model fit, (2) profile prevalence estimates, (3) item parameter estimates, (4) conditional response option probabilities, (5) attribute and profile classification agreement rates, and (6) individual continuous scores.

### Data

The dataset used in this study came from a survey of 8th grade students in Austria. We obtained this dataset from the “CDM” (Robitzsch et al., 2018) *R* package alongside the permission to use this dataset from the authors. In the survey, there were four questions asking about respondents' self-concept in math, and four questions asking about how much they enjoy studying math. Therefore, two attributes were specified: “math self-concept” and “math joy.” Each of the eight questions has four response options: 0 (low), 1 (mid-low), 2 (mid-high) and 3 (high). We randomly selected 500 individuals' responses from the entire dataset because we are interested in the model performance under small and attainable sample size conditions. We display the item-trait relationship and frequencies of each response option on each item in Table 2. A brief look of Table 2 reveals that the response data is positively skewed for items 1–4 (i.e., measuring math self-concept) with more individuals selecting options 0 and 1, while it is negatively skewed for items 5–8 (i.e., measuring math joy) with more individuals selecting options 2 and 3.

**Table 2:** Item data information.

**Item**	**Dimension**	**0 (Low)**	**1 (Mid-low)**	**2 (Mid-high)**	**3 (High)**
1	Math Self-concept	154 (30.8%)	233 (46.6%)	94 (18.8%)	19 (3.8%)
2	Math Self-concept	203 (40.6%)	178 (35.6%)	92 (18.4%)	27 (5.4%)
3	Math Self-concept	237 (47.4%)	153 (30.6%)	65 (13.0%)	45 (9.0%)
4	Math Self-concept	105 (21.0%)	197 (39.4%)	145 (29.0%)	53 (10.6%)
5	Math Joy	13 (2.6%)	67 (13.4%)	196 (39.2%)	224 (44.8%)
6	Math Joy	31 (6.2%)	136 (27.2%)	191 (38.2%)	142 (28.4%)
7	Math Joy	97 (19.4%)	160 (32.0%)	147 (29.4%)	96 (19.2%)
8	Math Joy	73 (14.6%)	160 (32.0%)	155 (31.0%)	112 (22.4%)

### Analysis

Parameters were estimated through implementing Hamiltonian Monte Carlo (HMC) algorithms in Stan (Carpenter et al., 2016). HMC has been acclaimed for its estimation efficiency compared to Gibbs sampler and the Metropolis algorithm especially when complex models including DCMs are involved (e.g., Girolami and Calderhead, 2011; da Silva et al., 2017; Jiang and Skorupski, 2017; Jiang and Templin, 2018; Luo and Jiao, 2018). The Stan codes used in this study for estimating the ORDM and MORDM are provided in the Supplementary Material.

We used less informative priors in the HMC algorithms with *N*(0, 20) for each item parameter and *Dirichlet*(2) for each attribute profile. The priors are considered less informative because a large standard deviation (i.e., 20) produces a relatively flat-shaped normal distribution, and a conjugate Dirichlet distribution with all equivalent parameter values (e.g., 2,2,2,2) is approximately a uniform distribution. Using less informative priors are recommended in similar DCM studies such as Chen et al. (2018), Culpepper and Hudson (2018), and Jiang and Carter (2018).

For each model, we ran two Markov chains with random starting values. The total length of the HMC sample was 6,000, for which the first 2,000 iterations were discarded as burn-in. To assess whether the Markov Chains converged to a stationary distribution the same as a posterior distribution, we computed the multivariate Gelman-Rubin convergence statistic $\hat{R}$ proposed by Brooks and Gelman (1998). $\hat{R}$ smaller than 1.1 for each parameter is usually considered convergence (Gelman and Rubin, 1992; Junker et al., 2016). For each of the three models, we obtained all the $\hat{R}$ smaller than 1.1.

We successfully applied the constraints designed for the ORDM to both the NRDM and the ORDM, and applied the constraints for the MORDM to itself through specifying pseudo response option parameters such that

(10) $$\lambda_{z,m=m}=\lambda_{z,m=1}+{\lambda^{\prime }}_{z,m=2}+\ldots +{\lambda^{\prime }}_{z,m=m}\;\forall \;z,m.$$

with the constraint ${\lambda^{\prime }}_{0,m}\leq 0\;\forall \;m$ and ${\lambda^{\prime }}_{z,m}\geq 0\;\forall \;z\geq 1,m$.

For model fit assessment, we used the leave-one-out (LOO) cross-validation approach for Bayesian estimation to compute the expected log predictive density (ELPD) and LOO information criterion (LOOIC) for each model. As suggested in Gelman et al. (2014), Vehtari et al. (2017) and Yao et al. (2018), the LOOIC is preferred over traditional simpler indices such as the Akaike information criterion (AIC), Bayesian information criterion (BIC) and deviance information criterion (DIC). Note that research has been lacking on the performance of the LOOIC for assessing DCM model fit. Regarding other latent variable models, Revuelta and Ximénez (2017) found that the LOOIC perform poorly with multidimensional continuous latent variable models, despite its fully Bayesian nature and excellent performance with unidimensional IRT models (e.g., Luo and Al-Harbi, 2017).

### Results

We estimated 48, 32, and 22 item parameters for the NRDM, the ORDM and the MORDM, respectively. For this dataset, the ORDM was 33% smaller than the NRDM, and the MORDM was 54% smaller than the NRDM. For each parameter, we report the mean of the posterior distribution as the point estimate and the standard deviation of the posterior distribution to indicate the uncertainty around the mean estimate. We first examined the results on model fit indices and listed the ELPD and LOOIC estimates and standard errors for each model in Table 3. For each index, smaller values indicate better fit. Although both indices suggested better fit for the ORDM than the other two models, their differences relative to the scale of the standard error indicate that the three models did not fit significantly different from each other. In practice, one would probably either select the most parsimonious MORDM or the best fitting ORDM for further interpretations.

**Table 3:** Model fit information in the operational study.

	**NRDM**		**ORDM**		**MORDM**	
	**Estimate**	**Se**	**Estimate**	**Se**	**Estimate**	**Se**
ELPD	−9.5	2.1	−9.3	2.0	−9.7	1.6
LOOIC	19.1	4.2	18.7	3.9	19.3	3.3

Examining profile prevalence estimates provides further evidence about the similar performance of the three models. Table 4 lists the estimates and standard deviations of the profile prevalence. Each estimate represents the probability of an individual having an attribute profile at large. The estimates for the NRDM were very similar to the ORDM as the point-estimate differences between the models were smaller than 0.01 for every profile. The point-estimate differences between the NRDM and the MORDM were all smaller than 0.02 for every profile.

**Table 4:** Profile prevalence estimates and standard deviations under the NRDM, the ORDM and the MORDM in the operational study.

**Profile**	**NRDM**	**ORDM**	**MORDM**
(0,0)	0.346 (0.029)	0.351 (0.027)	0.351 (0.026)
(1,0)	0.084 (0.021)	0.074 (0.016)	0.105 (0.019)
(0,1)	0.147 (0.024)	0.156 (0.022)	0.125 (0.021)
(1,1)	0.424 (0.028)	0.419 (0.025)	0.418 (0.024)

We could also look into the similarities of the item parameter estimates. Tables 5–7 display the item parameter estimates and their standard deviations for the NRDM, the ORDM, and the MORDM, respectively. Remember that the estimated pseudo parameters can be transformed to real parameters using Equation 10. For example, the intercept parameter for response option 2 of item 1 under the MORDM can be obtained through $\lambda_{0,i}+\lambda_{0,m=1}+\lambda_{0,m=2^{\prime }}=5.834-6.204-2.871=-3.241$. Results show that the parameter estimates were similar across the three models. For example, the intercept estimates for response option 1 of item 1 were −0.423, −0.390, and −0.370, respectively for the NRDM, ORDM and MORDM.

**Table 5:** NRDM: item parameter estimates and standard deviations in the operational study.

	**λ_0, *i, m* = 1_**	**${\lambda^{\prime }}_{0,i,m=2}$**	**${\lambda^{\prime }}_{0,i,m=3}$**	**λ_1, *i, m* = 1_**	**${\lambda^{\prime }}_{1,i,m=2}$**	**${\lambda^{\prime }}_{1,i,m=3}$**
Item 1	−0.423 (0.187)	−10.710 (4.389)	−12.578 (4.827)	3.619 (0.601)	10.339 (4.380)	10.981 (4.817)
Item 2	−0.934 (0.195)	−2.265 (0.439)	−1.927 (0.294)	2.149 (0.310)	1.995 (0.482)	0.744 (0.686)
Item 3	−1.557 (0.148)	−6.900 (1.610)	−10.818 (4.956)	2.714 (0.347)	6.354 (2.600)	10.481 (4.949)
Item 4	0.269 (0.064)	−4.540 (1.582)	−3.491 (1.854)	3.886 (1.815)	5.319 (1.790)	2.507 (1.846)
Item 5	1.986 (0.202)	−0.036 (0.003)	−1.241 (0.216)	16.908 (5.130)	2.756 (0.501)	2.130 (0.247)
Item 6	1.347 (0.210)	−0.716 (0.166)	−2.305 (0.471)	17.927 (5.469)	2.780 (0.345)	2.322 (0.481)
Item 7	0.296 (0.156)	−1.850 (0.210)	−2.255 (0.835)	0.926 (0.340)	2.857 (0.362)	1.940 (0.858)
Item 8	0.642 (0.145)	−10.306 (4.748)	−11.038 (4.195)	18.139 (5.770)	12.357 (4.731)	10.716 (5.182)

**Table 6:** ORDM: item parameter estimates and standard deviations in the operational study.

	**λ_0, *i, m* = 1_**	**${\lambda^{\prime }}_{0,i,m=2}$**	**${\lambda^{\prime }}_{0,i,m=3}$**	**λ_1, *i*_**
Item 1	−0.390 (0.154)	−4.088 (0.500)	−5.321 (0.562)	3.735 (0.496)
Item 2	−0.834 (0.144)	−2.181 (0.282)	−3.127 (0.338)	1.971 (0.236)
Item 3	−1.533 (0.200)	−3.377 (0.335)	−3.194 (0.334)	2.841 (0.274)
Item 4	0.289 (0.144)	−2.180 (0.327)	−3.784 (0.540)	2.902 (0.454)
Item 5	1.991 (0.214)	−0.029 (0.020)	−1.352 (0.224)	2.290 (0.216)
Item 6	1.352 (0.211)	−0.670 (0.146)	−2.586 (0.269)	2.626 (0.229)
Item 7	0.071 (0.144)	−1.370 (0.185)	−2.297 (0.235)	2.039 (0.181)
Item 8	0.646 (0.141)	−10.395 (3.543)	−12.733 (4.500)	12.427 (4.498)

**Table 7:** MORDM: item parameter estimates and standard deviations in the operational study.

	**λ_0, *i*_**	**λ_0, *m* = 1_**	**${\lambda^{\prime }}_{0,i,m=2}$**	**${\lambda^{\prime }}_{0,i,m=3}$**	**λ_1, *i*_**
Item 1	5.834 (2.232)	−6.204 (3.227)	−2.871 (0.182)	−3.781 (0.185)	2.472 (0.148)
Item 2	5.141 (2.238)	*	*	*	2.512 (0.154)
Item 3	4.491 (2.245)	*	*	*	2.732 (0.145)
Item 4	6.424 (2.236)	*	*	*	3.200 (0.162)
Item 5	10.313 (4.794)	−7.893 (4.766)	−0.527 (0.091)	−2.111 (0.120)	3.262 (0.181)
Item 6	9.339 (4.756)	*	*	*	2.348 (0.139)
Item 7	7.948 (4.770)	*	*	*	1.622 (0.111)
Item 8	8.285 (4.808)	*	*	*	2.033 (0.117)

The cells with “^*^”indicates that it shares the same parameter with the cell above it.

**Figure 1:**
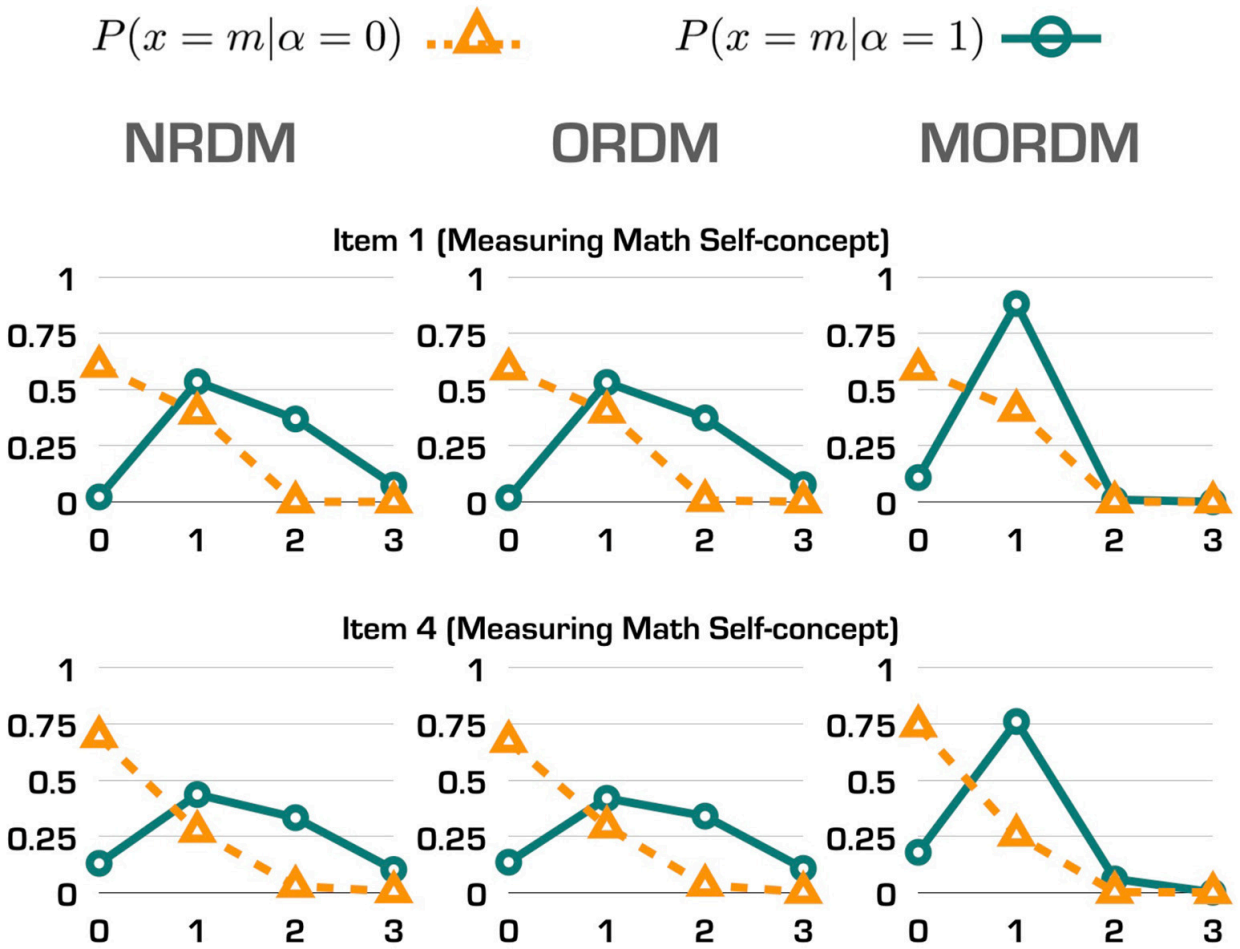
Response option curves for two items in the operational study.

Such similarities can be more revealing through computing probabilities of selecting each response option for individuals with and without the measured attribute. We selected items 1 and 4 (measuring math self-concept) to display their response option curves (ROCs) in Figure 1. The three ROCs for each item were similar to each other although those under the NRDM and the ORDM were even more alike. Also made clear by the ROCs is that the response option parameters in the MORDM are not unique to each item; instead, the first four items share the same set of response option parameters. Hence, the ROCs under the MORDM depart a bit more from those ROCs under the NRDM and the ORDM. The location of each intersection between the two curves on the *x*-axis in each graph represents the minimum response option where individuals with the attribute start to have higher probabilities to select than individuals without the attribute. For example, for items 1 and 4, individuals with the math self-concept have higher probabilities selecting response option 1 and above than those without the math self-concept.

**Figure 2:**
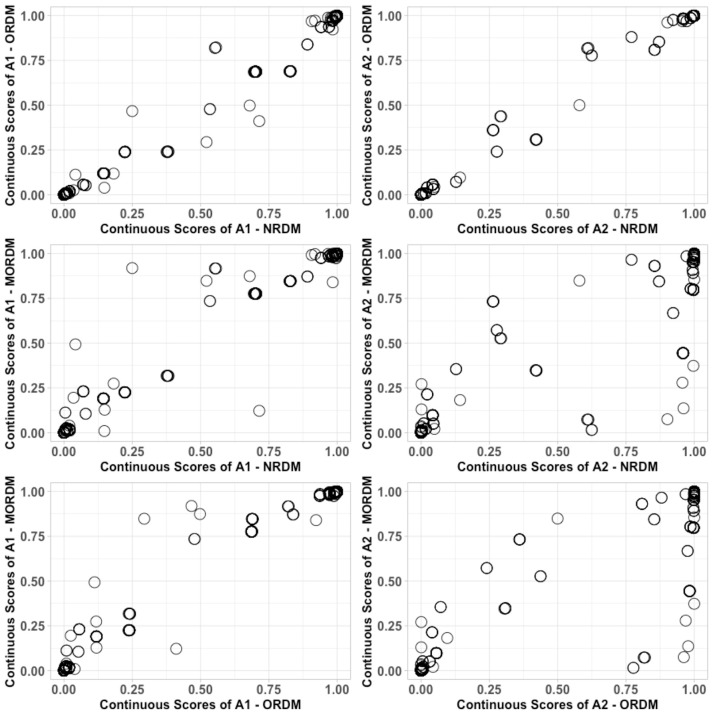
Comparison of continuous scores for each pair of models in the operational study.

Ultimately, the three models can be concluded to have similar performance if individuals have received similar categorical and continuous scores. The categorical scores include individuals' attribute and profile classifications. Table 8 cross-tabulates the attribute classification agreement between each pair of models. The agreement rates between the NRDM and the ORDM were very high: 99.0% and 99.8% for each attribute, respectively. The agreement rates were all over 99.0% on the math self-concept attribute for each pair of models, and the lowest agreement rate was on the math joy attribute: 92.6% between the ORDM and the MORDM. Table 9 cross-tabulates the profile classification agreement between each pair of models. Agreement rates between each pair were also very high. For example, only 6 out of the 500 individuals were classified into different profiles under the NRDM and the ORDM. The continuous scores are individuals' marginal probabilities of possessing each attribute (Liu et al., 2018). We display the continuous scores for all individuals between each pair of models in Figure 2. As expected, most individuals had scores close to either 0 or 1 under each model. For the pair of the NRDM and the ORDM, individuals' scores almost fit into a linear *y* = *x* line, meaning that both models produce very similar continuous scores. For other pairs, most scores can still fit into a linear line with only a few cases where scores differed substantially. To quantify the score differences, we computed the root-mean-square deviation (RMSD) for scores between each pair of models. For α_1_, the RMSD values were 0.04, 0.06 and 0.06 for NRDM/ORDM, NRDM/MORDM, and ORDM/MORDM, respectively. For α_2_, the RMSD values were 0.03, 0.15, and 0.16 for NRDM/ORDM, NRDM/MORDM, and ORDM/MORDM, respectively. To summarize, results show that the score differences were very small between the models, and we conclude that the three models performed similarly.

**Table 8:** Attribute possession agreement between each pair of models in the operational study.

**NRDM**	**ORDM**	
	α_1_ = 0	α_1_ = 0
α_1_ = 0	238 (47.6%)	0
α_1_ = 1	5 (1.0%)	257 (51.4%)
	α_2_ = 0	α_2_ = 1
α_2_ = 0	220 (44.0%)	0
α_2_ = 1	1 (0.2%)	279 (55.8%)
*The total number of agreements between the two models for α_1_ and α_2_ was 495 (99.0%) and 499 (99.8%), respectively. Cohen's Kappa for α_1_ and α_2_ were 0.98, and 1.00, respectively*.		
**NRDM**	**MORDM**	
	α_1_ = 0	α_1_ = 0
α_1_ = 0	237 (47.4%)	1 (0.2%)
α_1_ = 1	1 (0.2%)	261 (52.2%)
	α_2_ = 0	α_2_ = 1
α_2_ = 0	208 (41.6%)	12 (2.4%)
α_2_ = 1	24 (4.8%)	256 (51.2%)
*The total number of agreements between the two models for α_1_ and α_2_ was 498 (99.6%) and 464 (92.8%), respectively. Cohen's Kappa for α_1_ and α_2_ were 0.99, and 0.86, respectively*.		
**ORDM**	**MORDM**	
	α_1_ = 0	α_1_ = 0
α_1_ = 0	238 (47.6%)	5 (1.0%)
α_1_ = 1	0	257 (51.4%)
	α_2_ = 0	α_2_ = 1
α_2_ = 0	208 (41.6%)	13 (2.6%)
α_2_ = 1	24 (4.8%)	255 (51.0%)

*The total number of agreements between the two models for α_1_ and α_2_ was 495 (99.0%) and 463 (92.6%), respectively. Cohen's Kappa for α_1_ and α_2_ were 0.98, and 0.85, respectively*.

**Table 9:** Profile possession agreement between each pair of models in the operational study.

**NRDM**	**ORDM**			
	**(0,0)**	**(1,0)**	**(0,1)**	**(1,1)**
(0,0)	160 (32.0%)	0	0	0
(1,0)	3 (0.6%)	57 (11.4%)	0	0
(0,1)	1 (0.2%)	0	77 (15.4%)	0
(1,1)	0	0	2 (0.4%)	200 (40.0%)
*The total number of profile agreements between the two models was 494 (98.8%), with a Cohen's Kappa of 0.98*.				
**NRDM**	**MORDM**			
	**(0,0)**	**(1,0)**	**(0,1)**	**(1,1)**
(0,0)	151 (30.2%)	1 (0.2%)	8 (1.6%)	0
(1,0)	0	56 (11.2%)	0	4 (0.8)
(0,1)	8 (1.6%)	0	70 (14.0%)	0
(1,1)	0	16 (3.2%)	1 (0.2%)	185 (37.0%)
*The total number of profile agreements between the two models was 462 (92.4%), with a Cohen's Kappa of 0.89*.				
**ORDM**	**MORDM**			
	**(0,0)**	**(1,0)**	**(0,1)**	**(1,1)**
(0,0)	151 (30.2%)	4 (0.8%)	9 (1.8%)	0
(1,0)	0	53 (10.6%)	0	4 (0.8%)
(0,1)	8 (1.6%)	0	70 (14.0%)	1 (0.2%)
(1,1)	0	16 (3.2%)	0	184 (36.8%)

*The total number of profile agreements between the two models was 458 (91.6%), with a Cohen's Kappa of 0.88*.

## Simulation Study

### Methods

The purpose of this simulation study was to investigate whether the proposed ORDM and MORDM can provide unbiased parameter estimate and accurate attribute classification under the operational study condition. In order to do this, we used the parameters obtained from the operational study and generated 100 datasets in R (R Core Team, 2018) for each model. In each dataset, 500 individuals were simulated from a multinomial distribution of (0.351, 0.074, 0.156, 0.419) for each of the four attribute profiles: (0,0), (1,0), (0,1), and (1,1), respectively. We used the item parameters listed in Tables 6, 7 to generate item response for the ORDM and MORDM, respectively. We then fit the ORDM to its 100 datasets and the MORDM to its 100 datasets using the same HMC specifications in the operational study. Similar to what we did in the operational study to assess convergence, we obtained the multivariate Gelman-Rubin convergence statistic $\hat{R}$ and found that all the $\hat{R}$ values were between 0.97 and 1.01, indicating that the Markov chains have converged.

To assess parameter recovery, we computed the bias and root mean square error (RMSE) for each item parameter and attribute prevalence estimate. Bias and RMSE for parameter *x* were computed as:

(11) $$Bias(x)=\frac{\sum_{r=1}^{R}\left[{\hat{e}}_{r}(x)-e(x)\right]}{R},$$

(12) $$RMSE(x)=\sqrt{\frac{1}{R-1}{\sum_{r=1}^{R}\left[{\hat{e}}_{r}(x)-e(x)\right]}^{2}},$$

where *e*(*x*) is the true value of parameter *x*, ê_*r*_(*x*) is the *r*th replicate estimate of parameter *x* among *R* = 100 datasets. To assess classification accuracy, we explored the agreement between true and estimated classifications on each attribute and provided descriptive statistics on the agreement rates across the 100 datasets.

### Results

Tables 10, 11 list the bias and RMSE for the item parameter estimates in the ORDM and MORDM, respectively. Of interest is that most item parameter estimates list bias close to 0 and RMSE smaller than 0.5. We also observed that some of the biases and RMSEs are larger than others. For example, in Table 10, the bias and RMSE for λ_0, 5, *m* = 1_ seems larger than the λ_0, *i, m* = 1_ parameter for other items under the ORDM. We hypothesize that the unbalanced class membership probability and the uniqueness of the original distribution of examinee scores could both contribute to the larger bias and RMSE. A quick revisit of Table 2 reveals that the response option distribution of item 5 is negatively skewed, which sets itself apart from the other three items that measure the same attribute “math joy.” However, this is our initial hypothesis which may be test through a more robust simulation in the future.

**Table 10:** ORDM: bias and RMSE of estimated item parameters in the simulation study.

		**λ_**0, **i**, **m** = 1**_**	**${\lambda^{\prime }}_{0,i,m=2}$**	**${\lambda^{\prime }}_{0,i,m=3}$**	**λ_**1, **i_**
Bias	Item 1	0.009	−0.307	−0.344	0.308
	Item 2	−0.004	−0.021	−0.048	0.011
	Item 3	−0.047	−0.098	−0.090	0.098
	Item 4	−0.014	−0.115	−0.161	0.132
	Item 5	0.162	−0.101	−0.044	0.089
	Item 6	0.021	−0.020	−0.060	0.045
	Item 7	0.011	−0.015	−0.050	0.046
	Item 8	0.006	−0.431	−0.201	0.289
RMSE	Item 1	0.134	0.576	0.501	0.567
	Item 2	0.122	0.254	0.315	0.225
	Item 3	0.174	0.382	0.407	0.329
	Item 4	0.126	0.325	0.410	0.341
	Item 5	0.381	0.115	0.220	0.249
	Item 6	0.200	0.138	0.269	0.228
	Item 7	0.121	0.203	0.292	0.225
	Item 8	0.149	0.762	0.719	0.715

**Table 11:** MORDM: bias and RMSE of estimated item parameters in the simulation study.

		**λ_**0, **i_**	**λ_**0, **m** = 1**_**	**${\lambda^{\prime }}_{0,m=2}$**	**${\lambda^{\prime }}_{0,m=3}$**	**λ_**1, **i_**
Bias	Item 1	−0.176	0.168	−0.030	−0.039	0.033
	Item 2	−0.163	*	*	*	0.030
	Item 3	−0.207	*	*	*	0.031
	Item 4	−0.154	*	*	*	0.017
	Item 5	−0.172	0.312	0.009	−0.020	0.033
	Item 6	−0.202	*	*	*	0.019
	Item 7	−0.297	*	*	*	0.000
	Item 8	−0.269	*	*	*	0.009
RMSE	Item 1	0.468	0.471	0.162	0.178	0.152
	Item 2	0.491	*	*	*	0.159
	Item 3	0.509	*	*	*	0.156
	Item 4	0.488	*	*	*	0.176
	Item 5	0.175	0.450	0.086	0.127	0.164
	Item 6	0.458	*	*	*	0.137
	Item 7	0.437	*	*	*	0.109
	Item 8	0.410	*	*	*	0.134

The cells with “^*^” indicates that it shares the same parameter with the cell above it.

Table 12 displays the bias and RMSE for the attribute prevalence estimates in the ORDM and MORDM, respectively. All the bias and RMSE values in this table are smaller than 0.02. Table 13 contains the descriptive statistics for classification accuracy results. The classification accuracy for each attribute under both models are mostly above 0.99. Results show that both models can correctly recover parameters and provide accurate attribute classifications.

**Table 12:** Bias and RMSE of estimated attribute prevalence for the ORDM and MORDM in the simulation study.

**Profile**	**ORDM**		**MORDM**	
	**Bias**	**RMSE**	**Bias**	**RMSE**
(0,0)	−0.006	0.008	−0.004	0.009
(1,0)	0.000	0.005	0.000	0.007
(0,1)	0.020	0.021	0.018	0.022
(1,1)	−0.014	0.016	−0.014	0.017

**Table 13:** Descriptive statistics of attribute classification accuracy for the ORDM and MORDM in the simulation study.

	**ORDM**				**MORDM**			
	**Min**	**Mean**	**Max**	**SD**	**Min**	**Mean**	**Max**	**SD**
α_1_	0.992	0.998	1.000	0.002	0.981	0.995	1.000	0.005
α_2_	0.990	0.998	1.000	0.003	0.979	0.993	1.000	0.006

## Discussion

Scoring items in an ordinal fashion is common in educational and psychological tests. For example, an essay can be scored on a 0–6 scale, a two-step math question can be partially scored for responses on each step, and a questionnaire can have Likert-type items with eight response options. DCMs are psychometric models that aim to classify individuals into groups according to their estimated possession status of the measured attributes. Up to this point, polytomous DCMs, such as the NRDM and its special cases and extensions, are designed for nominal (i.e., unordered) responses. Although those DCMs can accommodate ordered response data, they ignore the monotonicity of response option probabilities and require a very large sample size to estimate. The ORDM and the MORDM were introduced in this paper to constrain the NRDM to situations where items are scored on an ordinal scale. Because the ORDM and the MORDM are polytomous extensions of the binary LCDM core, one could easily constrain the ORDM and the MORDM to arrive at other polytomous DCMs. For example, one could replace the LCDM core with the DINA model to arrive at the (modified) polytomous response DINA model.

The analysis of the survey data demonstrated that the proposed models perform similarly to the NRDM but with much fewer parameters to estimate. With four response options in this dataset, the ORDM was 33% smaller than the NRDM. The ORDM will show more comparative advantages if the number of response options increases. If there are seven response options, the ORDM requires estimations of 56 parameters, which is 42% smaller than the NRDM. The MORDM was 54% smaller than the NRDM in this dataset, and it will require only 29 item parameters if there are seven response options, which is 70% smaller than the NRDM. The smaller model sizes of the ORDM and the MORDM comparing to traditional polytomous models allow them to accommodate much smaller sample sizes and thus prove useful in many small-scale testing scenarios.

In addition to their smaller model sizes, the ORDM and the MORDM offer information that can easily capture item characteristics in addition to response option characteristics. In the NRDM, each type of parameters (i.e., intercept, main effects and interactions) is freely estimated for each response option on each item. As a result, it would be easier to discuss the quality of each response option than that of the whole item. In the ORDM, we only have one main effect parameter for each measured attribute representing its effect on the whole item. In the MORDM, we estimate a general intercept parameter: λ_0, *i*_ for each item, representing the general item difficulty. Such item-level information can be helpful for item selection, revision, and reporting.

We consider the study as one of the first steps incorporating the ordinal response option characteristics into DCMs. A major limitation of this study is that the findings are couched within the particular data used for this study. For future research, we encourage a more robust simulation study examining the performance of the ORDM and the MORDM under a wide range of factors. For example, one could examine the impact of sample sizes on the performance of the new models. We expect that both models can accommodate even smaller sample sizes than the dataset we used in this paper because DCMs, different from multidimensional item response theory models (e.g., Reckase, 1997), do not aim to precisely locate individuals on multiple continua. But this is unknown until tested. We also encourage researchers to investigate the impact of the Q-matrix complexity on the models' performance. Although the increase of Q-matrix complexity generally reduces model performance (e.g., Madison and Bradshaw, 2015; Liu et al., 2017), its impact on the ORDM and the MORDM remains unknown. In addition, we did not assume an ordered sequence on the possession of attributes in this study, although attribute structures can be found in educational and psychological assessment representing the presence of certain attributes given the presence/absence of other attributes (Leighton et al., 2004; Liu and Huggins-Manley, 2016; Liu, 2018). Examining the impact of different attribute structures on the model performance would be of interest. Finally, we used a fully Bayesian approach to estimate the model parameters. Alternatively, one could estimate the parameters via parametric approaches such as the expectation maximization (EM; e.g., Templin and Hoffman, 2013) and the differential evolution optimization (DEoptim; e.g., Jiang and Ma, 2018).

To summarize, the ORDM and the MORDM are psychometric models that can score ordinal item data to classify individuals into latent groups. They are much smaller and thus easier to implement than DCMs for nominal responses. They also offer useful item-level information in addition to option-level information. With the active research and practice in the area of diagnostic measurement, we anticipate that the proposed models will be useful for scoring polytomous item responses in a wide range of educational and psychological assessments.

## Author Contributions

All authors listed have made a substantial, direct and intellectual contribution to the work, and approved it for publication.

### Conflict of Interest Statement

The authors declare that the research was conducted in the absence of any commercial or financial relationships that could be construed as a potential conflict of interest.
