# Supplementary material for: Diagnostic Classification Models for Ordinal Item Responses
Source: Front Psychol. 2018 Dec 11;9:2512. doi: 10.3389/fpsyg.2018.02512 (PMC6297886; doi:10.3389/fpsyg.2018.02512)
Supplement: Supplementary file 1 [file Data_Sheet_1.docx]

Supplementary Material

**Sample Stan Code for the ORDM**

data{

int Np;

int Ni;

int Nc;

int Ns;

int Y[Np, Ni];

}

parameters{

simplex[Nc] Vc;

real<lower=0> l1_1;

real<lower=0> l2_1;

real<lower=0> l3_1;

real<lower=0> l4_1;

real<lower=0> l5_1;

real<lower=0> l6_1;

real<lower=0> l7_1;

real<lower=0> l8_1;

real l1_01;

real l2_01;

real l3_01;

real l4_01;

real l5_01;

real l6_01;

real l7_01;

real l8_01;

real<upper=0> l1_02;

real<upper=0> l2_02;

real<upper=0> l3_02;

real<upper=0> l4_02;

real<upper=0> l5_02;

real<upper=0> l6_02;

real<upper=0> l7_02;

real<upper=0> l8_02;

real<upper=0> l1_03;

real<upper=0> l2_03;

real<upper=0> l3_03;

real<upper=0> l4_03;

real<upper=0> l5_03;

real<upper=0> l6_03;

real<upper=0> l7_03;

real<upper=0> l8_03;

}

transformed parameters{

vector[Ns] PImat[Ni, Nc];

PImat[1,1,1]=0;

PImat[2,1,1]=0;

PImat[3,1,1]=0;

PImat[4,1,1]=0;

PImat[5,1,1]=0;

PImat[6,1,1]=0;

PImat[7,1,1]=0;

PImat[8,1,1]=0;

PImat[1,2,1]=0;

PImat[2,2,1]=0;

PImat[3,2,1]=0;

PImat[4,2,1]=0;

PImat[5,2,1]=0;

PImat[6,2,1]=0;

PImat[7,2,1]=0;

PImat[8,2,1]=0;

PImat[1,3,1]=0;

PImat[2,3,1]=0;

PImat[3,3,1]=0;

PImat[4,3,1]=0;

PImat[5,3,1]=0;

PImat[6,3,1]=0;

PImat[7,3,1]=0;

PImat[8,3,1]=0;

PImat[1,4,1]=0;

PImat[2,4,1]=0;

PImat[3,4,1]=0;

PImat[4,4,1]=0;

PImat[5,4,1]=0;

PImat[6,4,1]=0;

PImat[7,4,1]=0;

PImat[8,4,1]=0;

PImat[1,1,2]=l1_01;

PImat[2,1,2]=l2_01;

PImat[3,1,2]=l3_01;

PImat[4,1,2]=l4_01;

PImat[5,1,2]=l5_01;

PImat[6,1,2]=l6_01;

PImat[7,1,2]=l7_01;

PImat[8,1,2]=l8_01;

PImat[1,1,3]=l1_01+l1_02;

PImat[2,1,3]=l2_01+l2_02;

PImat[3,1,3]=l3_01+l3_02;

PImat[4,1,3]=l4_01+l4_02;

PImat[5,1,3]=l5_01+l5_02;

PImat[6,1,3]=l6_01+l6_02;

PImat[7,1,3]=l7_01+l7_02;

PImat[8,1,3]=l8_01+l8_02;

PImat[1,1,4]=l1_01+l1_02+l1_03;

PImat[2,1,4]=l2_01+l2_02+l2_03;

PImat[3,1,4]=l3_01+l3_02+l3_03;

PImat[4,1,4]=l4_01+l4_02+l4_03;

PImat[5,1,4]=l5_01+l5_02+l5_03;

PImat[6,1,4]=l6_01+l6_02+l6_03;

PImat[7,1,4]=l7_01+l7_02+l7_03;

PImat[8,1,4]=l8_01+l8_02+l8_03;

PImat[1,2,2]=l1_01+l1_1;

PImat[2,2,2]=l2_01+l2_1;

PImat[3,2,2]=l3_01+l3_1;

PImat[4,2,2]=l4_01+l4_1;

PImat[5,2,2]=l5_01;

PImat[6,2,2]=l6_01;

PImat[7,2,2]=l7_01;

PImat[8,2,2]=l8_01;

PImat[1,2,3]=l1_01+l1_1+l1_02+l1_1;

PImat[2,2,3]=l2_01+l2_1+l2_02+l2_1;

PImat[3,2,3]=l3_01+l3_1+l3_02+l3_1;

PImat[4,2,3]=l4_01+l4_1+l4_02+l4_1;

PImat[5,2,3]=l5_01+l5_02;

PImat[6,2,3]=l6_01+l6_02;

PImat[7,2,3]=l7_01+l7_02;

PImat[8,2,3]=l8_01+l8_02;

PImat[1,2,4]=l1_01+l1_1+l1_02+l1_1+l1_03+l1_1;

PImat[2,2,4]=l2_01+l2_1+l2_02+l2_1+l2_03+l2_1;

PImat[3,2,4]=l3_01+l3_1+l3_02+l3_1+l3_03+l3_1;

PImat[4,2,4]=l4_01+l4_1+l4_02+l4_1+l4_03+l4_1;

PImat[5,2,4]=l5_01+l5_02+l5_03;

PImat[6,2,4]=l6_01+l6_02+l6_03;

PImat[7,2,4]=l7_01+l7_02+l7_03;

PImat[8,2,4]=l8_01+l8_02+l8_03;

PImat[1,3,2]=l1_01;

PImat[2,3,2]=l2_01;

PImat[3,3,2]=l3_01;

PImat[4,3,2]=l4_01;

PImat[5,3,2]=l5_01+l5_1;

PImat[6,3,2]=l6_01+l6_1;

PImat[7,3,2]=l7_01+l7_1;

PImat[8,3,2]=l8_01+l8_1;

PImat[1,3,3]=l1_01+l1_02;

PImat[2,3,3]=l2_01+l2_02;

PImat[3,3,3]=l3_01+l3_02;

PImat[4,3,3]=l4_01+l4_02;

PImat[5,3,3]=l5_01+l5_1+l5_02+l5_1;

PImat[6,3,3]=l6_01+l6_1+l6_02+l6_1;

PImat[7,3,3]=l7_01+l7_1+l7_02+l7_1;

PImat[8,3,3]=l8_01+l8_1+l8_02+l8_1;

PImat[1,3,4]=l1_01+l1_02+l1_03;

PImat[2,3,4]=l2_01+l2_02+l2_03;

PImat[3,3,4]=l3_01+l3_02+l3_03;

PImat[4,3,4]=l4_01+l4_02+l4_03;

PImat[5,3,4]=l5_01+l5_1+l5_02+l5_1+l5_03+l5_1;

PImat[6,3,4]=l6_01+l6_1+l6_02+l6_1+l6_03+l6_1;

PImat[7,3,4]=l7_01+l7_1+l7_02+l7_1+l7_03+l7_1;

PImat[8,3,4]=l8_01+l8_1+l8_02+l8_1+l8_03+l8_1;

PImat[1,4,2]=l1_01+l1_1;

PImat[2,4,2]=l2_01+l2_1;

PImat[3,4,2]=l3_01+l3_1;

PImat[4,4,2]=l4_01+l4_1;

PImat[5,4,2]=l5_01+l5_1;

PImat[6,4,2]=l6_01+l6_1;

PImat[7,4,2]=l7_01+l7_1;

PImat[8,4,2]=l8_01+l8_1;

PImat[1,4,3]=l1_01+l1_1+l1_02+l1_1;

PImat[2,4,3]=l2_01+l2_1+l2_02+l2_1;

PImat[3,4,3]=l3_01+l3_1+l3_02+l3_1;

PImat[4,4,3]=l4_01+l4_1+l4_02+l4_1;

PImat[5,4,3]=l5_01+l5_1+l5_02+l5_1;

PImat[6,4,3]=l6_01+l6_1+l6_02+l6_1;

PImat[7,4,3]=l7_01+l7_1+l7_02+l7_1;

PImat[8,4,3]=l8_01+l8_1+l8_02+l8_1;

PImat[1,4,4]=l1_01+l1_1+l1_02+l1_1+l1_03+l1_1;

PImat[2,4,4]=l2_01+l2_1+l2_02+l2_1+l2_03+l2_1;

PImat[3,4,4]=l3_01+l3_1+l3_02+l3_1+l3_03+l3_1;

PImat[4,4,4]=l4_01+l4_1+l4_02+l4_1+l4_03+l4_1;

PImat[5,4,4]=l5_01+l5_1+l5_02+l5_1+l5_03+l5_1;

PImat[6,4,4]=l6_01+l6_1+l6_02+l6_1+l6_03+l6_1;

PImat[7,4,4]=l7_01+l7_1+l7_02+l7_1+l7_03+l7_1;

PImat[8,4,4]=l8_01+l8_1+l8_02+l8_1+l8_03+l8_1;

}

model {

vector[Nc] contributionsC;

vector[Ni] contributionsI;

//Prior

l1_1~normal(0,20);

l2_1~normal(0,20);

l3_1~normal(0,20);

l4_1~normal(0,20);

l5_1~normal(0,20);

l6_1~normal(0,20);

l7_1~normal(0,20);

l8_1~normal(0,20);

l1_01~normal(0,20);

l2_01~normal(0,20);

l3_01~normal(0,20);

l4_01~normal(0,20);

l5_01~normal(0,20);

l6_01~normal(0,20);

l7_01~normal(0,20);

l8_01~normal(0,20);

l1_02~normal(0,20);

l2_02~normal(0,20);

l3_02~normal(0,20);

l4_02~normal(0,20);

l5_02~normal(0,20);

l6_02~normal(0,20);

l7_02~normal(0,20);

l8_02~normal(0,20);

l1_03~normal(0,20);

l2_03~normal(0,20);

l3_03~normal(0,20);

l4_03~normal(0,20);

l5_03~normal(0,20);

l6_03~normal(0,20);

l7_03~normal(0,20);

l8_03~normal(0,20);

Vc~dirichlet(rep_vector(2.0, Nc));

//Likelihood

for (iterp in 1:Np){

for (iterc in 1:Nc){

for (iteri in 1:Ni){

contributionsI[iteri]= categorical_lpmf(Y[iterp,iteri]+1| softmax(((PImat[iteri,iterc]))));

}

contributionsC[iterc]=log(Vc[iterc])+sum(contributionsI);

}

target+=log_sum_exp(contributionsC);

}

}

**Sample Stan Code for the MORDM**

data{

int Np;

int Ni;

int Nc;

int Ns;

int Y[Np, Ni];

}

parameters{

simplex[Nc] Vc;

real<lower=0> l1_1;

real<lower=0> l2_1;

real<lower=0> l3_1;

real<lower=0> l4_1;

real<lower=0> l5_1;

real<lower=0> l6_1;

real<lower=0> l7_1;

real<lower=0> l8_1;

real l1_01;

real l2_01;

real l3_01;

real l4_01;

real l5_01;

real l6_01;

real l7_01;

real l8_01;

real<upper=0> step1D1;

real<upper=0> step2D1;

real<upper=0> step3D1;

real<upper=0> step1D2;

real<upper=0> step2D2;

real<upper=0> step3D2;

}

transformed parameters{

vector[Ns] PImat[Ni, Nc];

PImat[1,1,1]=0;

PImat[2,1,1]=0;

PImat[3,1,1]=0;

PImat[4,1,1]=0;

PImat[5,1,1]=0;

PImat[6,1,1]=0;

PImat[7,1,1]=0;

PImat[8,1,1]=0;

PImat[1,2,1]=0;

PImat[2,2,1]=0;

PImat[3,2,1]=0;

PImat[4,2,1]=0;

PImat[5,2,1]=0;

PImat[6,2,1]=0;

PImat[7,2,1]=0;

PImat[8,2,1]=0;

PImat[1,3,1]=0;

PImat[2,3,1]=0;

PImat[3,3,1]=0;

PImat[4,3,1]=0;

PImat[5,3,1]=0;

PImat[6,3,1]=0;

PImat[7,3,1]=0;

PImat[8,3,1]=0;

PImat[1,4,1]=0;

PImat[2,4,1]=0;

PImat[3,4,1]=0;

PImat[4,4,1]=0;

PImat[5,4,1]=0;

PImat[6,4,1]=0;

PImat[7,4,1]=0;

PImat[8,4,1]=0;

PImat[1,1,2]=l1_01+step1D1;

PImat[2,1,2]=l2_01+step1D1;

PImat[3,1,2]=l3_01+step1D1;

PImat[4,1,2]=l4_01+step1D1;

PImat[5,1,2]=l5_01+step1D2;

PImat[6,1,2]=l6_01+step1D2;

PImat[7,1,2]=l7_01+step1D2;

PImat[8,1,2]=l8_01+step1D2;

PImat[1,1,3]=l1_01+step1D1+step2D1;

PImat[2,1,3]=l2_01+step1D1+step2D1;

PImat[3,1,3]=l3_01+step1D1+step2D1;

PImat[4,1,3]=l4_01+step1D1+step2D1;

PImat[5,1,3]=l5_01+step1D2+step2D2;

PImat[6,1,3]=l6_01+step1D2+step2D2;

PImat[7,1,3]=l7_01+step1D2+step2D2;

PImat[8,1,3]=l8_01+step1D2+step2D2;

PImat[1,1,4]=l1_01+step1D1+step2D1+step3D1;

PImat[2,1,4]=l2_01+step1D1+step2D1+step3D1;

PImat[3,1,4]=l3_01+step1D1+step2D1+step3D1;

PImat[4,1,4]=l4_01+step1D1+step2D1+step3D1;

PImat[5,1,4]=l5_01+step1D2+step2D2+step3D2;

PImat[6,1,4]=l6_01+step1D2+step2D2+step3D2;

PImat[7,1,4]=l7_01+step1D2+step2D2+step3D2;

PImat[8,1,4]=l8_01+step1D2+step2D2+step3D2;

PImat[1,2,2]=l1_01+step1D1+l1_1;

PImat[2,2,2]=l2_01+step1D1+l2_1;

PImat[3,2,2]=l3_01+step1D1+l3_1;

PImat[4,2,2]=l4_01+step1D1+l4_1;

PImat[5,2,2]=l5_01+step1D2;

PImat[6,2,2]=l6_01+step1D2;

PImat[7,2,2]=l7_01+step1D2;

PImat[8,2,2]=l8_01+step1D2;

PImat[1,2,3]=l1_01+step1D1+step2D1+l1_1+l1_1;

PImat[2,2,3]=l2_01+step1D1+step2D1+l2_1+l2_1;

PImat[3,2,3]=l3_01+step1D1+step2D1+l3_1+l3_1;

PImat[4,2,3]=l4_01+step1D1+step2D1+l4_1+l4_1;

PImat[5,2,3]=l5_01+step1D2+step2D2;

PImat[6,2,3]=l6_01+step1D2+step2D2;

PImat[7,2,3]=l7_01+step1D2+step2D2;

PImat[8,2,3]=l8_01+step1D2+step2D2;

PImat[1,2,4]=l1_01+step1D1+step2D1+step3D1+l1_1+l1_1+l1_1;

PImat[2,2,4]=l2_01+step1D1+step2D1+step3D1+l2_1+l2_1+l2_1;

PImat[3,2,4]=l3_01+step1D1+step2D1+step3D1+l3_1+l3_1+l3_1;

PImat[4,2,4]=l4_01+step1D1+step2D1+step3D1+l4_1+l4_1+l4_1;

PImat[5,2,4]=l5_01+step1D2+step2D2+step3D2;

PImat[6,2,4]=l6_01+step1D2+step2D2+step3D2;

PImat[7,2,4]=l7_01+step1D2+step2D2+step3D2;

PImat[8,2,4]=l8_01+step1D2+step2D2+step3D2;

PImat[1,3,2]=l1_01+step1D1;

PImat[2,3,2]=l2_01+step1D1;

PImat[3,3,2]=l3_01+step1D1;

PImat[4,3,2]=l4_01+step1D1;

PImat[5,3,2]=l5_01+step1D2+l5_1;

PImat[6,3,2]=l6_01+step1D2+l6_1;

PImat[7,3,2]=l7_01+step1D2+l7_1;

PImat[8,3,2]=l8_01+step1D2+l8_1;

PImat[1,3,3]=l1_01+step1D1+step2D1;

PImat[2,3,3]=l2_01+step1D1+step2D1;

PImat[3,3,3]=l3_01+step1D1+step2D1;

PImat[4,3,3]=l4_01+step1D1+step2D1;

PImat[5,3,3]=l5_01+step1D2+step2D2+l5_1+l5_1;

PImat[6,3,3]=l6_01+step1D2+step2D2+l6_1+l6_1;

PImat[7,3,3]=l7_01+step1D2+step2D2+l7_1+l7_1;

PImat[8,3,3]=l8_01+step1D2+step2D2+l8_1+l8_1;

PImat[1,3,4]=l1_01+step1D1+step2D1+step3D1;

PImat[2,3,4]=l2_01+step1D1+step2D1+step3D1;

PImat[3,3,4]=l3_01+step1D1+step2D1+step3D1;

PImat[4,3,4]=l4_01+step1D1+step2D1+step3D1;

PImat[5,3,4]=l5_01+step1D2+step2D2+step3D2+l5_1+l5_1+l5_1;

PImat[6,3,4]=l6_01+step1D2+step2D2+step3D2+l6_1+l6_1+l6_1;

PImat[7,3,4]=l7_01+step1D2+step2D2+step3D2+l7_1+l7_1+l7_1;

PImat[8,3,4]=l8_01+step1D2+step2D2+step3D2+l8_1+l8_1+l8_1;

PImat[1,4,2]=l1_01+step1D1+l1_1;

PImat[2,4,2]=l2_01+step1D1+l2_1;

PImat[3,4,2]=l3_01+step1D1+l3_1;

PImat[4,4,2]=l4_01+step1D1+l4_1;

PImat[5,4,2]=l5_01+step1D2+l5_1;

PImat[6,4,2]=l6_01+step1D2+l6_1;

PImat[7,4,2]=l7_01+step1D2+l7_1;

PImat[8,4,2]=l8_01+step1D2+l8_1;

PImat[1,4,3]=l1_01+step1D1+step2D1+l1_1+l1_1;

PImat[2,4,3]=l2_01+step1D1+step2D1+l2_1+l2_1;

PImat[3,4,3]=l3_01+step1D1+step2D1+l3_1+l3_1;

PImat[4,4,3]=l4_01+step1D1+step2D1+l4_1+l4_1;

PImat[5,4,3]=l5_01+step1D2+step2D2+l5_1+l5_1;

PImat[6,4,3]=l6_01+step1D2+step2D2+l6_1+l6_1;

PImat[7,4,3]=l7_01+step1D2+step2D2+l7_1+l7_1;

PImat[8,4,3]=l8_01+step1D2+step2D2+l8_1+l8_1;

PImat[1,4,4]=l1_01+step1D1+step2D1+step3D1+l1_1+l1_1+l1_1;

PImat[2,4,4]=l2_01+step1D1+step2D1+step3D1+l2_1+l2_1+l2_1;

PImat[3,4,4]=l3_01+step1D1+step2D1+step3D1+l3_1+l3_1+l3_1;

PImat[4,4,4]=l4_01+step1D1+step2D1+step3D1+l4_1+l4_1+l4_1;

PImat[5,4,4]=l5_01+step1D2+step2D2+step3D2+l5_1+l5_1+l5_1;

PImat[6,4,4]=l6_01+step1D2+step2D2+step3D2+l6_1+l6_1+l6_1;

PImat[7,4,4]=l7_01+step1D2+step2D2+step3D2+l7_1+l7_1+l7_1;

PImat[8,4,4]=l8_01+step1D2+step2D2+step3D2+l8_1+l8_1+l8_1;

}

model {

vector[Nc] contributionsC;

vector[Ni] contributionsI;

//Prior

l1_1~normal(0,20);

l2_1~normal(0,20);

l3_1~normal(0,20);

l4_1~normal(0,20);

l5_1~normal(0,20);

l6_1~normal(0,20);

l7_1~normal(0,20);

l8_1~normal(0,20);

l1_01~normal(0,20);

l2_01~normal(0,20);

l3_01~normal(0,20);

l4_01~normal(0,20);

l5_01~normal(0,20);

l6_01~normal(0,20);

l7_01~normal(0,20);

l8_01~normal(0,20);

step1D1~normal(0,20);

step2D1~normal(0,20);

step3D1~normal(0,20);

step1D2~normal(0,20);

step2D2~normal(0,20);

step3D2~normal(0,20);

Vc~dirichlet(rep_vector(2.0, Nc));

//Likelihood

for (iterp in 1:Np){

for (iterc in 1:Nc){

for (iteri in 1:Ni){

contributionsI[iteri]= categorical_lpmf(Y[iterp,iteri]+1| softmax(((PImat[iteri,iterc]))));

}

contributionsC[iterc]=log(Vc[iterc])+sum(contributionsI);

}

target+=log_sum_exp(contributionsC);

}

}
